# Infectious Bursal Disease Virus Genotypic Diversity from Poultry in Latin America

**DOI:** 10.3390/v18070746

**Published:** 2026-07-06

**Authors:** Nilo Ikuta, Diéssy Kipper, André Salvador Kazantzi Fonseca, Vagner Ricardo Lunge

**Affiliations:** 1 Simbios Biotecnologia, Cachoeirinha 94940-030, RS, Brazil; ikuta@simbios.com.br (N.I.); kipper.simbios@gmail.com (D.K.); fonseca@simbios.com.br (A.S.K.F.); 2 Institute of Biotechnology, University of Caxias do Sul (UCS), Caxias do Sul 95070-560, RS, Brazil

**Keywords:** IBDV, America Latina, genogroups, genotypes, phylogenetic

## Abstract

Infectious bursal disease virus (IBDV) is a pathogen that causes Gumboro disease in young chickens. Vaccine strains and field IBDV genotypes are disseminated in chickens from commercial poultry farms worldwide. This study aimed to detect the field IBDV genotypic diversity in poultry farms in Latin America, mainly in Brazil. Bursal samples from 69 broiler flocks in eleven Latin American countries were obtained between 2015 and 2025. All 69 samples tested were positive for IBDV; the *VP2* (segment A) and *VP1* (segment B) genes were sequenced. Phylogenetic and amino acid substitution analyses were performed with large genetic datasets, including previously identified IBDV genotypes worldwide. The results revealed four A (A1, A2, A3, and A4) and three B (B1, B2, and the candidate B6) genogroups in Latin America. Furthermore, genotypes A1B1 (1.4%), A2B1 (59.4%), A3B2 (20.3%), A3B6 (2.9%), and A4B1 (15.9%) were identified. A2B1 could be subdivided into A2aB1a (24.4%), A2bB1a (29.3%), A2dB1b (19.5%), and A2eB1a (26.8%). In Brazil, the field genotypes A3B2, A4B1, and A3B6 were demonstrated. These findings highlight an important IBDV genotypic diversity in Latin American countries and reinforce the need for continuous molecular surveillance to support control and vaccination programs.

## 1. Introduction

Gumboro disease is a significant pathology affecting young chicks, including broilers and layers, caused by infectious bursal disease virus (IBDV) [1]. This virus exhibits a marked tropism for the bursa of Fabricius cells, leading to lymphocyte depletion, immunosuppression, and a substantial increase in susceptibility to secondary infections and immunization failures [2]. Clinical manifestations range from subclinical infections to outbreaks with high morbidity and mortality in poultry flocks, depending on different main factors such as the age and immune status of the birds, as well as the pathogenicity of the IBDV infecting strain [1,3].

IBDV is a double-stranded RNA (dsRNA) viral particle belonging to the family *Birnaviridae*, species *Avibirnavirus gumboroense* [4]. In addition to the genus *Avibirnavirus*, this family comprises other genera, including *Aquabirnavirus*, *Blosnavirus*, *Dronavirus*, *Entomobirnavirus*, *Mambirnavirus*, *Ronavirus*, and *Telnevirus* [4,5]. The IBDV complete genome is segmented into two separate dsRNAs, A and B. Segment A is approximately 3260 bp in length and encodes four viral proteins (VPs): VP2, VP3, VP4, and VP5. VP2 and VP3 have structural functions and are produced from a primary viral polyprotein further cleaved by VP4, a protease released as an active enzyme. VP2 plays a central role in IBDV antigenicity and immune recognition, being directly associated with strain-specific immune responses and also immunization efficacy. This protein also contains a hypervariable region (HVR) that encompasses key surface-exposed loops and hydrophilic peaks, serving as the main determinant of IBDV epitopes and the primary target for neutralizing antibodies. Additionally, VP5 is a non-structural protein encoded by a gene that overlaps the coding region of VP2, contributing to the virulence of IBDV [5,6]. Segment B is approximately 2880 bp, with an ORF encoding only the VP1 protein-dependent RNA polymerase, which governs replication and contributes to viral fitness and adaptation [7,8].

Since the first recognition as an important pathogen, IBDV has been classified according to antigenic, virulence, and genetic characteristics. It was first divided into serotype 1, detected in chickens and associated with clinical disease, and serotype 2, present in turkeys and without clinical relevance [9]. Due to the importance of poultry production, IBDVs from serotype 1 have been more well-characterized by antigenicity evaluation and virulence profile, being historically classified into four main groups: classical virulent (cvIBDVs), antigenic variants (avIBDVs), very virulent (vvIBDVs), and divergent IBDVs (dIBDVs) [1,2].

The development of DNA/RNA viral detection methods enabled a pivotal advance in IBDV genetic analysis and identification in the 1990s. The first studies focused on the analysis of nucleotide and amino acid sequences of the HVR in the *VP2* gene, with the development of molecular methods (such as RT-PCR followed by RFLP analysis and nucleotide sequencing) to differentiate IBDV into molecular groups [10,11]. These methods classified cvIBDVs, avIBDVs, vvIBDVs, and dIBDVs into dozens of molecular groups used to identify IBDVs in poultry production systems in the early 2000s. Later, a revised system was established to classify IBDVs into seven main genetic groups (G1 to G7) based on molecular and phylogenetic analyses of the *VP2* HVR gene [6]. More recently, a further improvement in the classification system included the analysis of both segments, A and B (*VP2* and *VP1* genes) [12]. IBDVs are now classified into nine genogroups for segment A (A0 to A8) and five for segment B (B1 to B5). Notably, previously named genogroups G1 to G7 correspond to A1 to A7, respectively [6,12]. The combined analysis of genogroups A and B, could better classify IBDVs into at least 15 different genotypes, including 14 of serotype 1 (A1B1, A1B2, A1B3, A2B1, A3B2, A3B1, A3B3, A3B4, A3B5, A4B1, A5B6, A6B1, A7B3, and A8B3) and one of serotype 2 (A0B1) [12].

Immunization is the primary strategy for controlling IBDV worldwide. Early control strategies relied on the use of live attenuated vaccines derived from the cvIBDV strains (genogroup A1), which were used worldwide and could control the first field Gumboro disease challenges. With the emergence of the avIBDVs (genogroup A2), vvIBDVs (genogroup A3), and dIBDVs (genogroup A4) in the 1980s and 1990s, additional live attenuated strains were adopted as vaccines in commercial poultry production worldwide. New vaccine technologies were further developed later (such as immune complex and rHVT-VP2 vectored) and are also used today. However, live attenuated strains have still been continuously used to immunize commercial poultry flocks worldwide, and vaccine strains (as well as vaccine-derived field strains) are widespread in many poultry-producing countries [1,13,14].

IBDV endemicity in all regions with intensive commercial poultry production (with a high diversity of field variants) and the long-term use of different live vaccine strains have contributed to a complex epidemiological scenario characterized by the co-circulation of multiple viral lineages, including vaccine strains and field variants. Also, field viruses derived from vaccine strains have been reported, reinforcing the complex evolution in commercial poultry farms. Recombination and reassortment events can generate mosaic genomes and contribute to the emergence of novel and concerning field variants [15]. In Latin America, the epidemiological landscape closely mirrors that observed in other major poultry-producing regions worldwide, with slight variations in some countries. Specifically in Brazil, the expansion of the poultry industry associated with high technological standards (including intensive use of live IBDV vaccines of genogroup A1) shaped a complex scenario, with the emergence and persistence of a high diversity of IBDVs in the commercial poultry farms [11,16,17,18,19,20]. In other countries (such as Argentina, Chile, Ecuador, and Uruguay), co-circulation of classical strains (cvIBDVs) and variants also suggests a similar epidemiological scenario. Recent reports demonstrated the occurrence of avIBDVs, vvIBDVs, and dIBDVs, among others [21,22,23,24,25]. In addition, the identification of reassortant and newly emerging genogroups, some of which are closely related to strains circulating in other continents, underscores the role of viral evolution and transcontinental spread in shaping the current IBDV genotype landscape [26]. Therefore, this study aimed to identify the field IBDV genogroups and genotype diversity in Latin America, mainly in Brazil, between 2015 and 2025.

## 2. Materials and Methods

### 2.1. Clinical Samples and Vaccine Strains

Sixty-nine bursae samples were included in the study. They were obtained from broiler flocks with suspected Gumboro disease in 11 different countries in Latin America between August 2015 and September 2025. The poultry flocks were from Argentina (*n* = 6), Bolivia (*n* = 2), Brazil (*n* = 27), Chile (*n* = 2), Colombia (*n* = 5), Ecuador (*n* = 7), El Salvador (*n* = 1), Honduras (*n* = 2), Mexico (*n* = 6), Nicaragua (*n* = 3), and Peru (*n* = 8). These samples were obtained by convenience from an even larger number of samples that had been previously genotyped by a routine nested RT-PCR-RFLP procedure [11,19]. Importantly, bursae samples testing positive for IBDV genogroup A1 (suggestive of vaccine strains from cvIBDV) were excluded from further sequencing analysis, except for one sample from Ecuador. All samples were collected by veterinarians with prior authorization for international shipment of biological material, and animal care practices were followed throughout the study.

In general, each sample consisted of a pool of five bursae per flock. These bursae were macerated and dotted on FTA cards or not (tissue samples). FTA cards were sent via standard express services with prior authorization for national or international shipment of biological material. Tissue samples were transported in ice boxes and subsequently stored at −20 °C until molecular analyses. In the laboratory, a portion of each fragment was collected, and the material was jointly macerated to obtain a total volume of approximately 20 mg.

### 2.2. Viral RNA Extraction and RT-PCR

Viral RNA was extracted using the commercial NewGene^®^ Prep and PreAmp kits (Simbios Biotecnologia, Cachoeirinha, Brazil), following the manufacturer’s instructions. RT-PCR assays were carried out with the total extracted DNA using NewGene^®^ IBDVAmp master mix (Simbios Biotecnologia, Cachoeirinha, Brazil). All PCRs were performed in a Step One Plus™ Real-Time PCR System Thermal Cycler (Applied Biosystems, Norwalk, CT, USA) under the following conditions: denaturation at 95 °C for 3 min, followed by 40 cycles of 95 °C for 15 s and annealing/extension at 60 °C for 60 s. PCR amplification curves for all samples were evaluated in comparison with IBDV-positive controls. Reaction tubes without any DNA template were also included as a negative control in all independent runs. Samples were considered positive when presenting a characteristic amplification curve with a cycle threshold (Ct) value below 38 (Ct < 38).

### 2.3. Sequencing

The RT–PCR–positive samples were submitted for genotype confirmation using the NewGene IBDVSeq master mix (Simbios Biotecnologia, Cachoeirinha, Brazil) for amplification of the *VP1* and *VP2* genes. A nested RT-PCR (RT-nPCR) was performed according to a previously published protocol [12]. The amplified products were purified and submitted for Sanger sequencing. The *VP2* gene fragment corresponded approximately to nucleotide positions 496 to 1135, while the *VP1* gene fragment encompassed positions 208 to 888 of the respective genomic A and B segments. The obtained nucleotide sequences were assembled, analyzed, and deposited in the GenBank database (Table S1).

### 2.4. GenBank Data Collection

*VP2* and *VP1* gene sequences of reference strains from the five genera of the family *Birnaviridae* were obtained from GenBank, highlighting strain CEF94 (AF194428 and AF194429) of the genus *Avibirnavirus*, strain LCBV (MK103419 and MK103420) of the genus *Blosnavirus*, strain West Buxton (AF078668 and AF078669) of the genus *Aquabirnavirus*, strain DXV (U60650 and AF196645) of the genus *Entomobirnavirus,* and PBRV strain represented by GDFS9-2018 (MZ080605 and MZ080606) of the genus *Mambirnavirus*. Additionally, 372 *VP2* gene sequences and 330 *VP1* gene sequences of IBDV (*Avibirnavirus gumborense*) were also obtained from Genbank (Tables S2 and S3), and all were also included in the reference datasets previously published [12,27].

All downloaded sequences were trimmed to retain only the segment between the nucleotide positions 526–1104 of the *VP2* gene, corresponding to the HVR in the VP2 peptide chain (amino acids 176–368), and between nucleotide positions 235–780 of the *VP1* gene, corresponding to part of the N-terminal domain and part of the finger subdomain of the VP1 polymerase (amino acids 79–260) [12,27]. After that, these sequences were aligned with the here-sequenced *VP2* and *VP1* gene fragments using MAFFT v7 [28].

### 2.5. Phylogenetic and Amino Acid Substitution Analyses

A phylogenetic tree was reconstructed using the maximum likelihood (ML) method implemented in the W-IQ-TREE web server [29], the optimal nucleotide substitution model selected using ModelFinder v3.1.3 [30], and 1000 replicates of the ultrafast bootstrap approximation [31]. Branches with bootstrap support values ≥70% were considered reliable, as commonly adopted in phylogenetic analyses [32].

**Figure 1:**
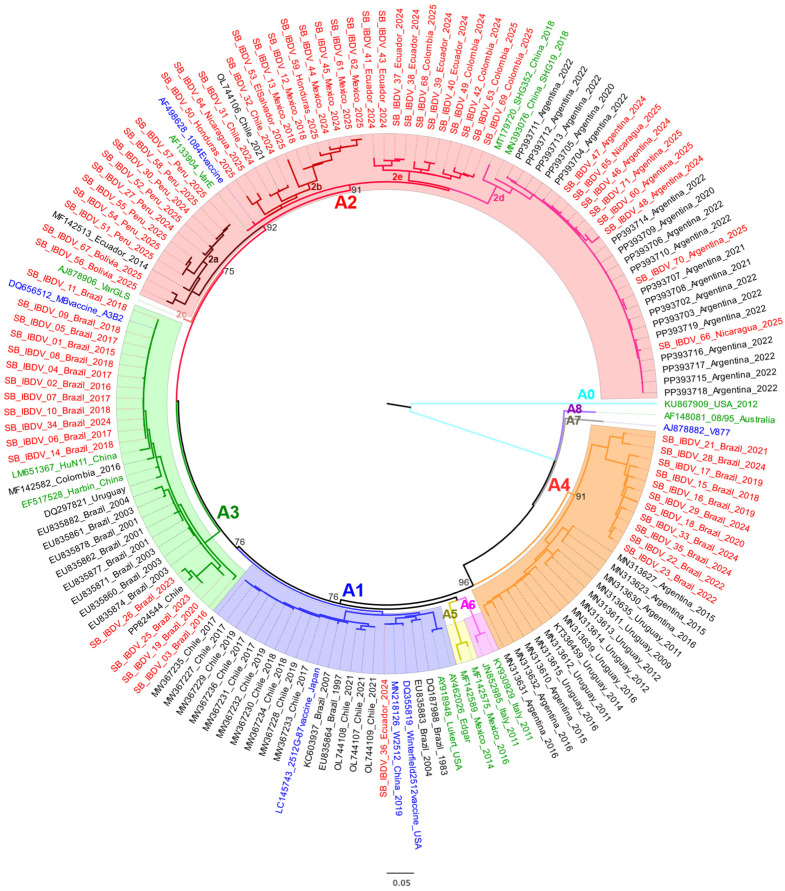
Maximum likelihood phylogeny inferred from *VP2* gene nucleotide sequences (*n* = 151) of IBDV. The dataset included 62 sequences from Latin America, 69 bursae sequences generated in this study (red labels), six reference vaccine strains (blue labels; detailed publicly available information is provided in Table S2), and 14 sequences representing genogroups A0, A1, A2, A3, A5, A6, A7, and A8 (green labels). Clade colors indicate *VP2* genogroups: blue = A1 (cvIBDV), red = A2 (avIBDV), green = A3 (vvIBDV), orange = A4 (dIBDV), yellow = A5 (Mexican), pink = A6 (Italian), gray = A7 (Early Australian), purple = A8 (Australian Variant) and light blue = A0 (Serotype 2). Major bootstrap values are indicated at the nodes.

**Figure 2:**
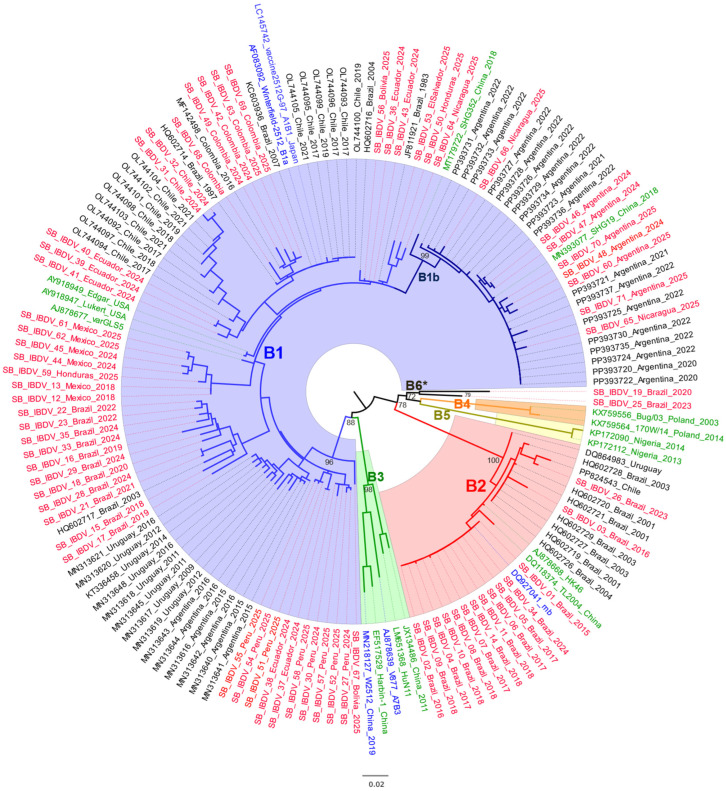
Maximum likelihood phylogeny inferred from *VP1* gene nucleotide sequences (*n* = 148) of IBDV. The dataset included 60 sequences from Latin America, 69 bursae sequences generated in this study (red labels), five reference vaccine strains (blue labels; detailed publicly available information is provided in Table S3), and 14 sequences representing genogroups B1, B2, B3, B4, and B5 (green labels). Clade colors indicate *VP1* genogroups: blue = B1 (cvIBDV-like), red = B2 (vvIBDV-like), green = B3 (Early Australian-like), orange = B4 (Polish and Tanzanian), yellow = B5 (Nigerian), and black = a candidate to a novel genogroup, tentatively designated B6 *. Major bootstrap values are indicated at the nodes.

For both genomic segments (A and B), two phylogenetic datasets were analyzed. The first dataset included all sequences generated in this study, all Latin American sequences retrieved, and representative reference strains from each genogroup to support genotype classification. These analyses were used to generate the phylogenetic trees presented in Figure 1 and Figure 2. The second dataset comprised all sequences included in the curated dataset for each genomic segment, regardless of geographic origin, and was used to reconstruct the comprehensive phylogenetic trees presented in the Supplementary Material (Figures S1 and S2).

Alignments of nucleotide and amino acid sequences were generated using MAFFT v. 7 [28]. Major substitutions were visually inspected in Geneious v. 2021.2.2 (Biomatters Ltd., Auckland, New Zealand; www.geneious.com). Nucleotide and amino acid sequences here obtained were compared with reference sequences representative of each genogroup. Additional pairwise identity analyses were performed to generate nucleotide and amino acid similarity matrices.

### 2.6. Genogroups and Genotypes Identification

Nucleotide phylogenetic analyses and trees for both segments were independently used to determine A and B genogroups. The robustness of phylogenetic trees and the identification of well-supported clades were supported by 1000 ultrafast bootstrap replicates generated using IQ-TREE version 3 [29,31]. IBDVs were further classified by the combination of segments A and B genotype, according to previously published work [12].

## 3. Results

### 3.1. IBDV Genogroups/Genotypes Identification

All 69 bursa samples from broiler chicken flocks with suspect Gumboro disease tested positive for IBDV by RT-PCR. Partial sequencing of the *VP2* and *VP1* genes was subsequently performed for all samples. The resulting *VP2* (segment A) and *VP1* (segment B) gene sequences were deposited in GenBank under accession numbers PZ380734–PZ380871 (Table S1).

Preliminary phylogenetic analyses using a few representative sequences of each genogroup suggested the occurrence of four A (A1, A2, A3, A4) and three B (B1, B2, B6) genogroups in the 69 field samples. In the analysis of A genogroups, A2 was the most prevalent (*n* = 41; 59.4%), followed by A3 (*n* = 16; 23.2%), A4 (*n* = 11; 15.9%), and A1 (*n* = 1; 1.4%). In the analysis of B genogroups, B1 (*n* = 53; 76.8%) was the most frequent, followed by B2 (*n* = 14; 20.3%) and B6 (*n* = 2; 2.9%). In addition, five main genotypes could be identified: A1B1, A2B1, A3B2, A3B6, and A4B1 (Table 1).

### 3.2. Phylogenetic and Amino Acid Substitution Analyses of the VP2 Gene

A more robust phylogenetic tree for segment A was constructed with the nucleotide sequences of the *VP2* gene, including reference sequences of the five genera of the family *Birnaviridae* (*Avibirnavirus*, *Blosnavirus*, *Aquabirnavirus*, *Entomobirnavirus*, and *Mambirnavirus*), 372 previously published IBDV data, and the 69 sequences generated in this study. The resulting topology revealed nine well-supported major clades (genogroups A0–A8, Figure S1). Within genogroup A2, five distinct subclades could also be observed (being identified as A2a, A2b, A2c, A2d, and A2e), which reflected additional genetic diversification within this genogroup. Comparison with previously proposed classification systems showed that the strains previously assigned to the G2a–G2d sublineages [6] correspond to the A2a–A2d genogroups [27,33], as well as to the genetic clustering observed in the present study (Table S2). Therefore, the additional and well-supported lineage identified here, which did not cluster within any of the previously recognized A2a–A2d subgenogroups, was designated as the novel subgenogroup A2e. The number and organization of the main A clades were consistent with those reported in previous studies [12,27,33].

The 69 Latin American IBDVs from broiler flocks sequenced here were again classified into four genogroups: A1, A2, A3, and A4. One IBDV was from genogroup A1 (a sample originated from Ecuador). A total of 41 IBDVs were classified within genogroup A2, including samples originating from Argentina (*n* = 6), Bolivia (*n* = 2), Chile (*n* = 2), Colombia (*n* = 5), Ecuador (*n* = 6), El Salvador (*n* = 1), Honduras (*n* = 2), Mexico (*n* = 6), Nicaragua (*n* = 3), and Peru (*n* = 8). Within this genogroup, four distinct subgenogroups were detected: A2a (*n* = 10), A2b (*n* = 12), A2d (*n* = 8), and the novel one, A2e (*n* = 11). Finally, 16 and 11 IBDVs clustered within genogroups A3 and A4, respectively, all detected in samples from Brazil (Table 1, Figure 1 and Figure S1).

The amino acid residues of the VP2 protein fragment were also evaluated (Table 2 and Table S4). Some key amino acid residues can characterize specific genogroups; for example, amino acid residues 249K and 286I are specific to A2; 222A, 242I, 256I, and 294I are specific to A3; and 222S, 289P, 290I, and 296F are specific to A4. In addition, A2a was characterized by residues 222Q and 254K, A2b by 318N, A2d by 252I, and A2e by residues 270E, 321V, and 325I, and A2e sequences shared an average amino acid identity of approximately 94% with other A2 subgenogroups and 97% among themselves (Table S5).

Among the genogroup A2, the sequences identified in this study clustered within the A2a, A2b, and A2d subgenogroups. The A2a and A2b subgenogroups include variant strains previously reported in the United States and other countries, whereas A2d comprises Chinese variant strains. No sequences from this study clustered within the A2c subgenogroup, which has been reported exclusively among North American strains [33]. Additionally, a distinct cluster composed of sequences from Ecuador and Colombia was identified. This cluster was consistently supported by phylogenetic analyses, unique amino acid signatures, and nucleotide identity comparisons, supporting the proposal of a novel subgenogroup, designated A2e (Figure 1 and Figure S1, Table S2).

### 3.3. Phylogenetic and Amino Acid Substitution Analyses of VP1

Based on the nucleotide sequences of partial *VP1* from the reference strain representatives of the five genera of the family *Birnaviridae* (*Avibirnavirus*, *Blosnavirus*, *Aquabirnavirus*, *Entomobirnavirus*, and *Mambirnavirus*), 330 previously published IBDV data, and the 69 sequences here generated, a phylogenetic tree was also constructed for segment B. The resulting topology revealed five well-supported major clades (genogroups B1–B5, Figure S2). Within genogroup B1, two subclades (B1a and B1b) were observed, reflecting further genetic diversification within this lineage. The number and organization of the main B clades were consistent with those reported in previous studies [12,27,33]. In addition, two IBDVs from Brazil clustered separately, suggesting an additional clade/genogroup (B6).

Therefore, the 69 Latin American IBDVs were classified into three B genogroups: B1 (*n* = 53), B2 (*n* = 14), and B6 (*n* = 2). IBDVs within genogroup B1 were detected in samples from Argentina (*n* = 6), Bolivia (*n* = 2), Brazil (*n* = 11), Chile (*n* = 2), Colombia (*n* = 5), Ecuador (*n* = 7), El Salvador (*n* = 1), Honduras (*n* = 2), Mexico (*n* = 6), Nicaragua (*n* = 3), and Peru (*n* = 8). All genogroup B2 and B6 samples were from Brazil. In addition, two distinct subclades were observed in sequences within the B1 genogroup: B1a (*n* = 45) and B1b (*n* = 8) (Table 1, Figure 2 and Figure S2).

The amino acid residues of the VP1 protein fragment were further evaluated. Importantly, B1 presented 145N, B2 had 146D, 147N, and 242E, and B6 was characterized by the residue 163V (Table 3 and Table S6). Furthermore, the two samples candidates of the B6 genogroup shared 99% amino acid identity and 93.0% nucleotide identity between themselves. When compared with the other segment B genogroups, these sample sequences exhibited an overall average nucleotide identity of 89.2%, with mean identities of 89.2%, 89.4%, 88.0%, 90.7%, and 89.0% relative to genogroups B1, B2, B3, B4, and B5, respectively (Tables S7 and S8).

### 3.4. Genotypic Diversity

The combined analysis of A and B genogroups here sequenced confirmed the occurrence of five genotypes in Latin America: A1B1, A2B1, A3B2, A3B6, and A4B1 (Table 1). Overall, the A2B1 combination was the most prevalent (*n* = 41; 59.4%), followed by A3B2 (*n* = 14; 20.3%), A4B1 (*n* = 11; 15.9%), A3B6 (*n* = 2; 2.9%) and A1B1 (*n* = 1; 1.4%). The geographic distribution of genotypes revealed distinct patterns across Latin America, with A2B1 showing the widest distribution, identified in Argentina, Bolivia, Chile, Colombia, Ecuador, El Salvador, Honduras, Mexico, Nicaragua, and Peru (but not in Brazil). In contrast, A3B2, A3B6, and A4B1 were detected exclusively in Brazil, highlighting a more restricted geographic distribution for these genotypes.

**Figure 3:**
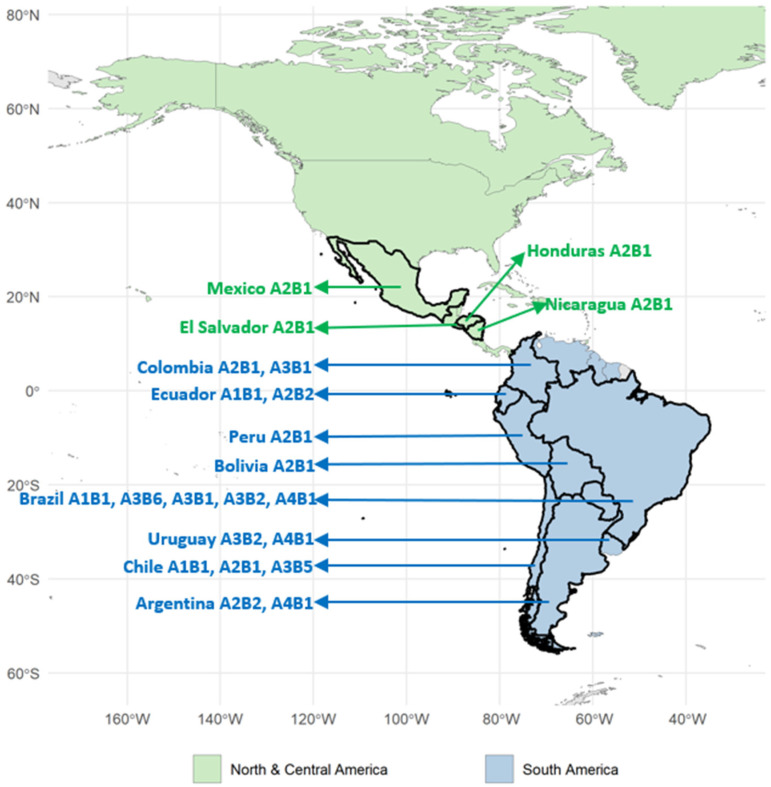
Geographic overview of Latin America showing the distribution of IBDV genotypes based on this study (*n* = 69; bursa), previous papers (*n* = 15), and public datasets (*n* = 46).

A final genotypic analysis was performed with all published data and sequences retrieved from public datasets, including 130 IBDVs from Latin America [16,22,23,25,26,34,35]. Among the identified genotype combinations, A2B1 was the most prevalent, accounting for 46.2% (*n* = 60) of the sequences and showing a broad geographical distribution across Colombia, Ecuador, Bolivia, Peru, El Salvador, Honduras, Mexico, Nicaragua, Chile, and Argentina, but not in Brazil. The second most frequent genotype was A4B1, comprising 19.2% (*n* = 25), being identified in Brazil, Argentina, and Uruguay. This was followed by A3B2, accounting for 16.9% (*n* = 22) and identified in Brazil and Uruguay. Next, A1B1 represented 13.8% (*n* = 18) and was reported in Ecuador, Chile, and Brazil. Less frequently detected genotypes included A3B6 (1.5%; *n* = 2), identified exclusively in Brazil; A3B1 (1.5%; *n* = 2), detected in Colombia and Brazil; and A3B5 (0.8%; *n* = 1), identified only in Brazil. Overall, Brazil exhibited the highest genotype diversity in the dataset, with the circulation of A1B1, A3B6, A3B1, A3B2, A3B5, and A4B1, while A2B1 remained the only genotype not detected in the country (Table 4 and Figure 3).

A2B1 subgenotypes were also evaluated. A2dB1b was the most prevalent (44%; *n* = 26), being identified in Argentina and Nicaragua, followed by A2bB1a (20%; *n* = 12), detected in El Salvador, Honduras, Mexico, Nicaragua, and Chile, the novel one A2eB1a (19%; *n*= 11), reported in Colombia and Ecuador, and A2aB1a (17%, *n* = 10), occurring in Bolivia and Peru. Overall, these findings indicate that A2B1 genotypes are broadly distributed across Latin America, with different subgenotypes, but not in Brazil (Table 5).

## 4. Discussion

This study performed the sequencing and phylogenetic analysis of 69 IBDVs obtained between 2015 and 2025, aiming to provide an overview of the genetic diversity of genogroups/genotypes circulating in the field in Latin America in recent years. In addition to samples from Brazil (the main poultry-producing country in the region), IBDVs from other poultry-producing regions of South, Central, and North America were also obtained and included to perform a more complete epidemiological analysis. The new information obtained here was also compared with 61 previously published reports from South America (Argentina, Brazil, Uruguay, and Colombia), resulting in an even larger dataset with 130 IBDVs from different bursa samples obtained in Latin America.

The separate phylogenetic evaluation of the IBDV A and B segment sequences obtained here (69 bursa samples from broiler flocks), with an additional 446 A segment (*VP2* gene) and 404 B segment (*VP1* gene) sequences, demonstrated tree topologies almost identical to those previously published [12,27,33]. In addition, four other genera from the family *Birnaviridae* (*Blosnavirus*, *Aquabirnavirus*, *Entomobirnavirus*, and *Mambirnavirus*) were included to reinforce the phylogenetic reconstructions. The resulting analyses consistently supported the clear clustering of IBDVs within the avian lineage, while maintaining a distinct separation from aquatic, insect, and mammalian-associated birnaviruses. Although mammalian-associated birnaviruses remain phylogenetically distant from classical avibirnaviruses, their identification raises important questions regarding potential host range expansion and the evolutionary history of the family *Birnaviridae* [5].

In the specific analysis of segment A, the 69 new IBDVs sequenced in this study were classified into genogroups A2 (*n* = 41; 59.4%), A3 (*n* = 16; %), A4 (*n* = 11; 15.9%), and A1 (*n* = 1; 1.4%). All IBDVs belonging to genogroup A2 from Latin America clustered with other IBDVs of the same group (i.e., avIBDV) from the USA and were distributed throughout South, Central, and North America, in agreement with previous reports [12,33]. Despite the wide dissemination in the Americas, IBDV belonging to the A2 genogroup has been rarely identified in Brazil to date. Interestingly, the dominant A genogroups in this important poultry-producing country are A3 and A4. A3 genogroup (i.e., vvIBDV) has been detected in Brazil for a long time [19]. Previous studies have shown that it was introduced in the 1990s and was previously associated with severe outbreaks characterized by high mortality rates [11,16]. Over time, the clinical impact of these viruses has become less severe, probably due to natural exposure and the widespread adoption of vaccination strategies against these specific viruses in commercial poultry farms. Nevertheless, the continued detection of A3 strains in Brazil suggests that this genogroup remains actively circulating in the country. In addition, A4 (i.e., dIBDV) has also been frequently identified in Brazil [11]. This lineage emerged early in the evolutionary history of IBDV and was introduced to South America probably in the 1960s. It diversified into independent subpopulations in Brazil, Argentina, and Uruguay through migration routes [19,34,35,36,37,38]. In Brazil, A4 IBDVs have been circulating for decades and have become the predominant variants in poultry farms. The widespread circulation of A4 strains in Brazil may also be contributing to limiting the establishment or spread of other potentially more concerning genogroups, such as A2 and even A3, possibly through population-level immunity and competitive viral dynamics in flocks of poultry on commercial farms.

A1 genogroup was detected in only one sample, which originated from Ecuador. As expected, this single A1 virus sequenced in this study clustered with other IBDVs from this genogroup (i.e., cvIBDV), as also previously described [12,34]. However, the frequency of this genogroup is likely much higher in commercial broiler production flocks due to the intensive use of live attenuated vaccines with A1 strains, as well as field strains derived from these vaccines and even other A1 IBDVs that may be circulating on poultry farms in these countries. It is important to reiterate that bursa samples exhibiting this pattern, mainly originating from Brazil, were excluded in a previous screening using a routine nested RT-PCR-RFLP procedure [11,19]. Therefore, the genotypic frequencies presented here should not be interpreted as exact representative estimates of the prevalence of all circulating IBDV genogroups in the sampled poultry-producing regions of Latin America.

A2 genogroup identified in this study could be subdivided into the A2a, A2b, A2d, and A2e subgenogroups. The A2a and A2b subgenogroups include variant strains previously reported in the United States and other countries, whereas A2d comprises Chinese variant strains. No sequences from this study clustered within the A2c subgenogroup, which has been reported exclusively among North American strains [33]. Additionally, a distinct cluster composed of sequences from Ecuador and Colombia was consistently supported by phylogenetic analyses, unique amino acid signatures, and nucleotide identity comparisons, supporting the proposal of a novel subgenogroup, designated A2e.

Additionally, VP2 amino acid sequences were evaluated to identify molecular signatures characteristic of each genogroup. Overall, amino acid sequence patterns were identified, highlighting 249K and 286I in A2, A222, I242, I256, and I294 in A3, and 289P, 290I, and 296F in A4. These findings are also in agreement with previous studies demonstrating that distinct IBDV genogroups possess some specific amino acid signatures that are useful for molecular classification and epidemiological tracking [12,34,36].

Although classification of IBDV has historically relied mainly on the analysis of segment A, the additional evaluation of segment B has been proposed in recent years [12,27]. Here, the 69 IBDVs sequenced were classified into genogroups B1 (*n* = 53; 76.8%), B2 (*n* = 14; 20.3%), and B6 (*n* = 2; 2.9%). This very high B1 frequency was already expected, since nucleotide sequences characteristic of this genogroup were already identified frequently combined with three prevalent A genogroups: A1 (cvIBDVs), A2 (avIBDVs), and A4 (dIBDVs) [12,27]. In fact, all IBDVs from genogroups A1, A2, and A4 sequenced in this study clustered in the B1 clade in the phylogenetic tree. In contrast, IBDVs from genogroup A3 presented two different sequence types of the B segment: B2 and a candidate for a novel one, here named as B6. The two B6 IBDVs (SB_IBDV_25 and SB_IBDV_19) were identified from bursal samples collected in Brazil in 2020 and 2023. Despite being detected in different years, they clustered together and formed a distinct phylogenetic lineage clearly separated from all previously recognized segment B genogroups (B1–B5). In addition, they displayed a unique amino acid profile that did not fully correspond to any known IBDV lineage. The two sequences shared 93.0% nucleotide identity with each other, while exhibiting an overall mean nucleotide identity of approximately 89.2% relative to the established B1–B5 genogroups, corresponding to a divergence close to the threshold proposed for the recognition of novel segment B genogroups [12]. Although these findings support a novel segment B lineage, here tentatively designated B6, its formal classification requires additional evidence, as the currently available data comprise only two sequences and therefore do not fulfill the minimum sampling criteria proposed for the definitive establishment of a new genogroup.

The B1b IBDV sequences presented characteristic amino acid signatures at positions 141I, 147D, 163V, and 260E in VP1, which have previously been reported as markers of the B1b genogroup [33]. These residues were also found in the Chinese SHG19 strain, a representative B1b virus [33]. In addition, pairwise sequence comparisons showed that the Argentinean and Nicaraguan B1b strains of this study shared 98.7% nucleotide identity and 99.1% amino acid identity with SHG19, further supporting their close genetic relationship (Table S9). Therefore, the association between these South American strains and SHG19 is supported not only by shared amino acid signatures characteristic of B1b viruses, but also by the high overall sequence identity observed among these strains [26,27]. In addition, the B2 genogroup was characterized by the conserved amino acid markers 146D, 147N, and 242E as previously reported [34], while B6 was characterized by the residue 163V. Taken together, these results reinforce the occurrence of B genogroup-specific amino acid signatures, supporting their usefulness for further molecular characterization and epidemiological surveillance of circulating IBDVs.

Furthermore, the combined analysis of segments A and B provides a more comprehensive understanding of viral diversity, evolutionary dynamics, and rearrangement events circulating in different geographic regions. All combinations of genogroups A and B observed in this study allowed the 69 field samples to be classified into five genotypes (A1B1, A2B1, A3B2, A3B6, and A4B1), while the inclusion of data from other studies (totaling 130 Latin American IBDVs) resulted in seven IBDV genotypes (adding A3B1 and A3B5 to the other five). Curiously, A3 was identified as having a high diversity of B segments across four genotypes (A3B1, A3B2, A3B3, and A3B6) in samples from Latin America [16,25]. In contrast, A1, A2, and A4 are always combined with B1 as previously reported [16,22,23,26,35].

To better understand the dissemination of the highly diverse A2B1 genotype, analyses of the respective circulating subgenotypes in Latin America were also carried out in this study. In general, A2dB1b was the most prevalent (*n* = 26; 44%), but this frequency was strongly influenced by previously published data from Argentina [26]. The new data published here demonstrated a more complex scenario regarding this genotype, with the identification of two previously reported subgenotypes: A2bB1a (*n* = 12; 20%), detected in El Salvador, Honduras, Mexico, Nicaragua, and Chile, and A2aB1a (*n* = 10; 17%), present in Bolivia and Peru. In addition, the new subgenotype A2eB1a (*n* = 11; 19%) was identified in samples from Colombia and Ecuador. In summary, these findings indicate that A2B1 genotypes are diverse and distributed throughout Latin America, with the exception of Brazil. These data will be important for tracking these highly disseminating A2B1 subgenotypes, as previously demonstrated for A2dB1b, which was initially identified in China but has rapidly spread to Middle Eastern and South American countries. Its recent detection in South America, particularly in Argentina, provides strong evidence of transcontinental dissemination [26,33]. Fortunately, this genotype has not yet been reported in Brazil, but its occurrence in neighboring countries underscores the need for enhanced surveillance and preventive measures to monitor its potential introduction and spread.

## 5. Conclusions

In conclusion, high genotypic diversity of infectious bursal disease virus (IBDV) was observed in commercial poultry farms in Latin America. Four A genogroups (A1, A2, A3, and A4) and three B genogroups (B1, B2, and B6) were identified, and their combined analysis revealed five distinct genotypes: A1B1, A2B1, A3B2, A3B6, and A4B1. Notably, the A2B1 genotype exhibited high intragenotypic diversity, including A2aB1a, A2bB1a, A2dB1b, and A2eB1a. In Brazil, the occurrence of the field genotypes A3B2 and A4B1 was confirmed, as well as the identification of the new genotype A3B6. Overall, these results highlight the considerable genetic diversity and dynamic patterns of IBDV across this important poultry-producing continent, reinforcing the need for continuous molecular surveillance to establish control strategies and optimize vaccination programs.

## Figures and Tables

**Table 1:** Genogroups and genotypes of the 69 field IBDVs analyzed in this study.

Segment A Genogroup (*n*)	Segment B Genogroup (*n*)	Genotype (*n*)	Country (*n*)
A1 (1)	B1 (1)	A1B1 (1)	Ecuador (1)
A2 (41)	B1 (41)	A2B1 (41)	Argentina (6), Bolivia (2), Chile (2), Colombia (5), Ecuador (6), El Salvador (1), Honduras (2), Mexico (6), Nicaragua (3), Peru (8)
A3 (16)	B2 (14)	A3B2 (14)	Brazil (14)
	B6 * (2)	A3B6 (2)	Brazil (2)
A4 (11)	B1 (11)	A4B1 (11)	Brazil (11)

* B6 is a candidate to a novel genogroup.

**Table 2:** Variable amino acid residues in the VP2 protein of IBDV genogroups. Key amino acid substitutions among the A genogroups are highlighted in red and bold. Amino acid positions in the VP2 protein are shown at the top. The complete analysis is presented in Table S4.

	222	242	249	256	258	264	269	270	279	286	289	290	294	296	305	317	321	324	325
A1	P	V	Q	A/V	D/G	I	T	T	D/N	I/T	L	M	L	I	I	S	A	Q	M
A2	T	V	**K**	V	G	I	T	A/E	N	**I**	L	M	L	I	I	S	A/V	Q	M
A3	**A**	**I**	Q	**I**	G	I	T	A	D/N	T	L	M	**I**	I	I	S	A	Q	M
A4	**S**	V	Q	V	G	I	T	T	N	T	**P**	**I**	L	**F**	I	S	A/T	Q	M
A5	T	V	Q	V	G	I	T	**K**	N	T	L	M	L	I	I	**K**	**P**	Q	M
A6	Q	V	Q	**K**	D	I	**S**	T	D	T	L	M	L	I	I	S	V	Q	M
A7	P	V	Q	V	**N**	**V**	A/T	T	**G**	T	L	M	L	I	**V**	S	A	Q	M
A8	A	V	Q	A	D	I	T	T	D	T	L	M	L	V	I	**R**	A	**E**	M
A0	P	V	Q	**E**	D	I	T	E	D	T	L	**V**	L	V	I	S	A	**P**	**I**

**Table 3:** Variable amino acid residues in the VP1 protein of IBDV genogroups. Key amino acid substitutions among the six B genogroups are highlighted in red and bold. Amino acid positions in the VP1 protein are shown at the top. The complete analysis is presented in Table S6.

	145	146	147	163	219	242
B1	**N**	E	G	A	D	D
B2	T	**D**	**N**	A	D	**E**
B3	T	E	S	A	D	D
B4	S	E	G	A	E	D
B5	Q	E	G	A/V	E	D
B6 *	T	E	S	**V**	E	D

* B6 a candidate to a novel genogroup.

**Table 4:** Comparative distribution of IBDV genotypes in Latin America according to this study (*n* = 69) and previously published articles and sequences in databases (*n* = 61).

Genotype	This Study *n* (%)	Countries	Dataset and Other Studies *n* (%)	Countries	Total *n* (%)	Countries
A1B1	1 (1.5)	Ecuador	17 (27.9)	Brazil, Chile	18 (13.8)	Brazil, Chile, Ecuador
A2B1	41 (59.4)	Argentina, Bolivia, Chile, Colombia, Ecuador, El Salvador, Honduras, Mexico, Nicaragua, Peru	19 (31.1)	Argentina, Chile	60 (46.2)	Argentina, Bolivia, Chile, Colombia, Ecuador, El Salvador, Honduras, Mexico, Nicaragua, Peru
A3B6 *	2 (2.9)	Brazil	0 (0)	NR	2 (1.5)	Brazil
A3B1	0 (0)	NR	2 (3.3)	Colombia, Brazil	2 (1.5)	Colombia, Brazil
A3B2	14 (20.3)	Brazil	8 (13.1)	Brazil, Uruguay	22 (17)	Brazil, Uruguay
A3B5	0 (0)	NR	1 (1.6)	NR	1 (0.8)	Chile
A4B1	11 (15.9)	Brazil	14 (23)	Argentina, Uruguay	25 (19.2)	Brazil, Argentina, Uruguay
Total	69 (100)		61 (100)		130 (100)	

NR—Not reported; * B6 a candidate to a novel genogroup.

**Table 5:** Comparative distribution of IBDV within the A2B1 genotypes in Latin America based on this study (*n* = 69, bursae) and previous papers (*n* = 18).

Genotype	This Study *n* (%)	Previous Studies *n* (%)	Total *n* (%)	Countries
A2eB1a	11 (26.8)	0 (0)	11 (19)	Colombia, Ecuador
A2aB1a	10 (24.4)	0 (0)	10 (17)	Bolivia, Peru
A2bB1a	12 (29.3)	0 (0)	12 (20)	Chile, El Salvador, Honduras, Mexico, Nicaragua
A2dB1b	8 (19.5)	18 (100)	26 (44)	Argentina, Nicaragua
Total	41 (100)	18 (100)	59 (100)	

## Data Availability

The data presented in this study are openly available in the NCBI under accession numbers PZ380734–PZ380875. Additional data supporting the findings of this study are available from the corresponding author upon reasonable request.

## Supplementary Materials

The following supporting information can be downloaded at: https://www.mdpi.com/article/10.3390/v18070746/s1, Figure S1: Maximum likelihood phylogeny based on nucleotide *VP2* gene sequences of IBDV (*n* = 446). Red labels highlight the 69 sequences generated in this study, while blue labels indicate vaccine reference strains. Clade colors denote genogroups: blue = A1 (cvIBDV), red = A2 (avIBDV), green = A3 (vvIBDV), orange = A4 (dIBDV), yellow = A5 (Mexican), pink = A6 (Italian), gray = A7 (Early Australian), and light blue = A0 (Serotype 2). Representatives of the five genera within the family *Birnaviridae* were included as references: *Avibirnavirus* strain CEF94, *Blosnavirus* strain LCBV, *Aquabirnavirus* strain West Buxton, *Entomobirnavirus* strain DXV, and *Mambirnavirus* strain GDFS9-2018. Bootstrap support values for the main clades are indicated at the corresponding nodes; Figure S2: Maximum likelihood phylogeny based on nucleotide *VP1* gene sequences of IBDV (*n* = 404). Red labels highlight the 69 sequences generated in this study, while blue labels indicate vaccine reference strains. Clade colors denote genogroups: blue = B1 (cvIBDV-like; B1b sequences are indicated by circles), red = B2 (vvIBDV-like), green = B3 (early Australian-like), orange = B4 (Polish and Tanzanian), yellow = B5 (Nigerian), and black = a candidate to a novel genogroup, here designated as B6 *. Representatives of the five genera within the family *Birnaviridae* were included as references: *Avibirnavirus* strain CEF94, *Blosnavirus* strain LCBV, *Aquabirnavirus* strain West Buxton, *Entomobirnavirus* strain DXV, and *Mambirnavirus* strain GDFS9-2018. The strain SHG19 (MN393077), identified in China, is highlighted in blue. Bootstrap support values for the main clades are indicated at the corresponding nodes; Table S1: Metadata and accession information for 69 IBDV sequences generated in this study; Table S2: Metadata and accession information for 372 IBDV VP2 reference sequences; Table S3: Metadata and accession information for 330 IBDV VP1 reference sequences; Table S4: Amino acid residues in the VP2 protein of IBDV genogroups identified in sequences generated in this study and representative sequences from different genogroups. Key amino acid substitutions among genogroups are shown in yellow/orange. Dots indicate identity with the reference sequence. Amino acid positions are indicated at the top; Table S5: Pairwise amino acid identity (%) of the VP2 protein among IBDV genotypes, including sequences generated in this study and representative reference strains. Colors denote different genotypes; Table S6: Amino acid residues in the VP1 protein of IBDV genogroups identified in sequences generated in this study and representative sequences from different genogroups. Key amino acid substitutions among genogroups are shown in yellow/orange. Dots indicate identity with the reference sequence. Amino acid positions are indicated at the top; Table S7: Pairwise amino acid identity (%) of the VP1 protein among IBDV genotypes, including sequences generated in this study and representative reference strains. Colors denote different genotypes; Table S8: Pairwise nucleotide identity (%) of the VP1 gene among IBDV genotypes, including sequences generated in this study and representative reference strains. Colors denote different genotypes; Table S9: VP1 nucleotide and amino acid polymorphisms, and pairwise sequence identity (%) and difference matrices among B1b sequences. Dots indicate nucleotide or amino acid residues identical to the consensus sequence.
