# Supplementary figures and images for: Infectious Bursal Disease Virus Genotypic Diversity from Poultry in Latin America
Source: Viruses. 2026 Jul 6;18(7):746. doi: 10.3390/v18070746 (PMC13431511; doi:10.3390/v18070746)

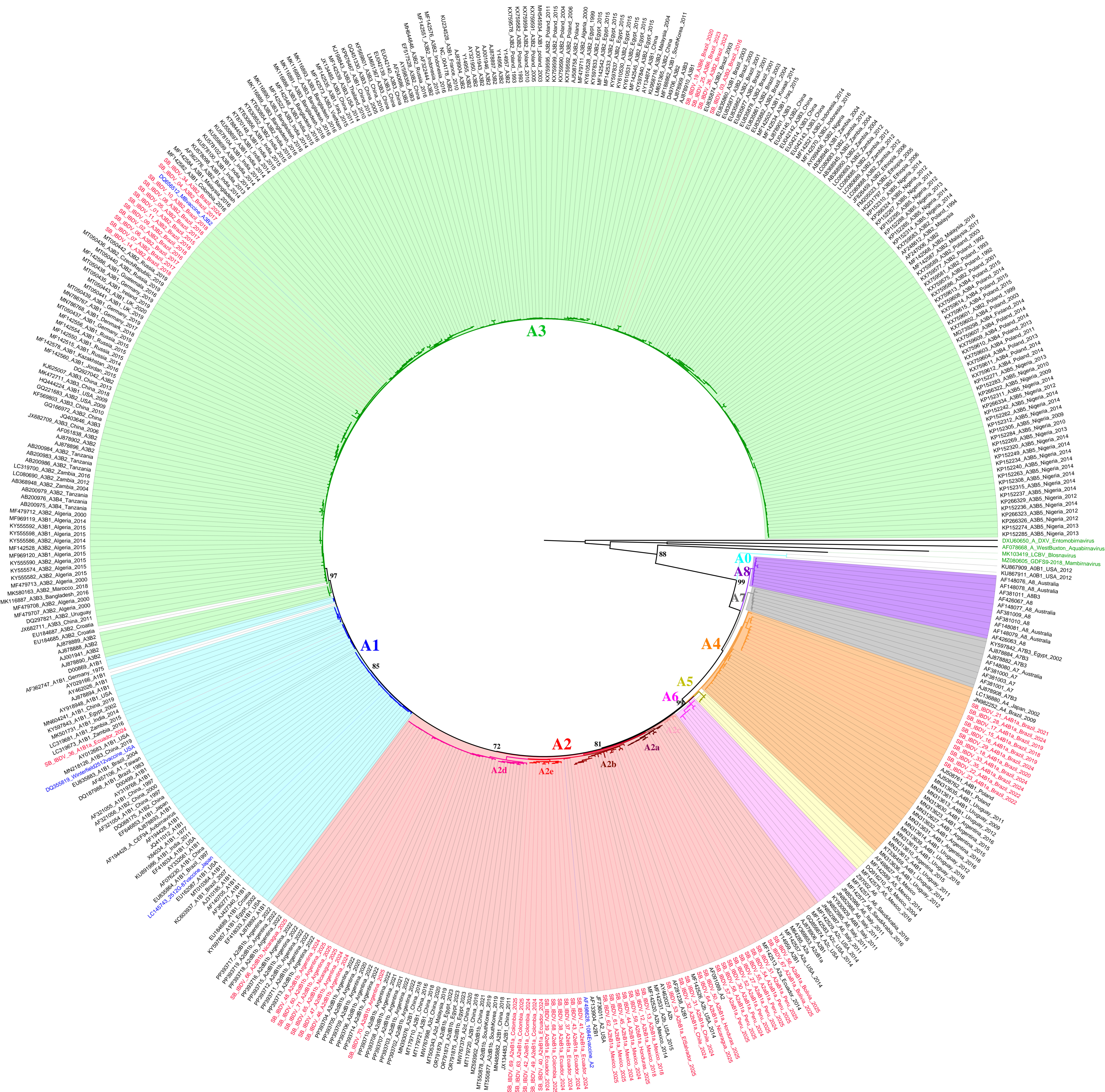

Supplement: Supplementary file 1 [file viruses-18-00746-s001.zip › viruses-4364260-supplementary-updated/Figure S1.pdf]

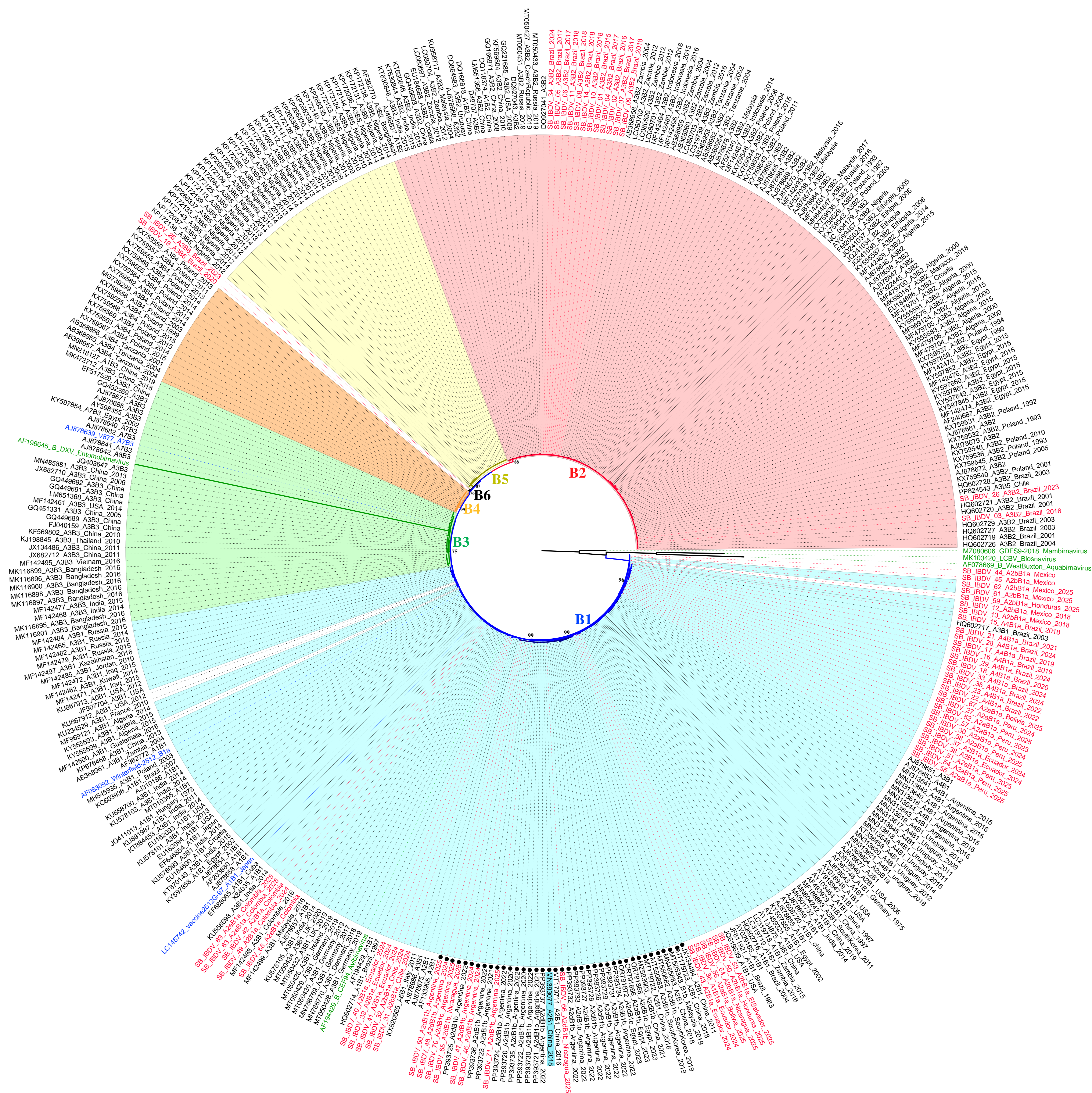

Supplement: Supplementary file 1 [file viruses-18-00746-s001.zip › viruses-4364260-supplementary-updated/Figure S2.pdf]
